# A privileged intraphagocyte niche is responsible for disseminated infection of *Staphylococcus aureus* in a zebrafish model

**DOI:** 10.1111/j.1462-5822.2012.01826.x

**Published:** 2012-07-04

**Authors:** Tomasz K Prajsnar, Ruth Hamilton, Jorge Garcia-Lara, Gareth McVicker, Alexander Williams, Michael Boots, Simon J Foster, Stephen A Renshaw

**Affiliations:** 1 Krebs Institute, University of SheffieldWestern Bank, Sheffield, S10 2TN, UK; 2 Department of Molecular Biology and Biotechnology, University of SheffieldWestern Bank, Sheffield, S10 2TN, UK; 3 MRC Centre for Developmental and Biomedical Genetics, University of SheffieldWestern Bank, Sheffield, S10 2TN, UK; 4 Department of Animal and Plant Sciences, University of SheffieldWestern Bank, Sheffield, S10 2TN, UK; 5 Department of Infection and Immunity, University of SheffieldBeech Hill Road, Sheffield, S10 2RX, UK; 6 Centre for Ecology and Conservation, University of Exeter, Cornwall CampusPenryn, TR10 9EZ, UK

## Abstract

The innate immune system is the primary defence against the versatile pathogen, *Staphylococcus aureus*. How this organism is able to avoid immune killing and cause infections is poorly understood. Using an established larval zebrafish infection model, we have shown that overwhelming infection is due to subversion of phagocytes by staphylococci, allowing bacteria to evade killing and found foci of disease. Larval zebrafish coinfected with two *S. aureus* strains carrying different fluorescent reporter gene fusions (but otherwise isogenic) had bacterial lesions, at the time of host death, containing predominantly one strain. Quantitative data using two marked strains revealed that the strain ratios, during overwhelming infection, were often skewed towards the extremes, with one strain predominating. Infection with passaged bacterial clones revealed the phenomenon not to bedue to adventitious mutations acquired by the pathogen. After infection of the host, all bacteria are internalized by phagocytes and the skewing of population ratios is absolutely dependent on the presence of phagocytes. Mathematical modelling of pathogen population dynamics revealed the data patterns are consistent with the hypothesis that a small number of infected phagocytes serve as an intracellular reservoir for *S. aureus*, which upon release leads to disseminated infection. Strategies to specifically alter neutrophil/macrophage numbers were used to map the potential subpopulation of phagocytes acting as a pathogen reservoir, revealing neutrophils as the likely ‘niche’. Subsequently in a murine sepsis model, *S. aureus* abscesses in kidneys were also found to be predominantly clonal, therefore likely founded by an individual cell, suggesting a potential mechanism analogous to the zebrafish model with few protected niches. These findings add credence to the argument that *S. aureus* control regimes should recognize both the intracellular as well as extracellular facets of the *S. aureus* life cycle.

## Introduction

*Staphylococcus aureus* is a leading cause of fatal bacteraemia, with mortality rates in UK hospitals reaching 30% (Thwaites *et al*., 2011). The route of human infection for *S. aureus* bacteraemia is mostly unknown, but is widely assumed to be via skin puncture – either accidental or often iatrogenic. The mechanisms by which this leads to bacteraemia and fatality are not well defined, but there is increasing evidence that phagocytes play an important role in disseminating disease (Thwaites and Gant, 2011). Multiple lines of evidence from human and animal studies suggest that phagocytes, and particularly neutrophils, may provide an intracellular niche for the dissemination of *S. aureus* during infection (Rogers and Tompsett, 1952; Rogers, 1956; 1959; Rogers and Melly, 1960; Gresham *et al*., 2000; Kubica *et al*., 2008; Thwaites and Gant, 2011).

It is widely known that mycobacteria generate an intracellular niche in which they can evade host killing, and recent work has shown how phagocytes can act to disseminate infection, rather than controlling it (Davis and Ramakrishnan, 2009). The key experiments which defined the role of the macrophage in disseminating mycobacterial infection were performed in larval zebrafish, where the ability to visualize host–phagocyte interaction *in vivo* and to study both host and pathogen genetics combine to generate new insights into vertebrate immunity (Renshaw and Trede, 2012). We have previously established a model of *S. aureus* bacteraemia in larval zebrafish, which recapitulates many important features of mammalian *S. aureus* models. In particular, host survival is phagocyte dependent, a large inoculum is required, and infection results in the appearance of foci akin to abscesses (Prajsnar *et al*., 2008). Using defined bacterial mutants, several virulence determinants were identified, including the regulator *saeR*. By combining bacterial mutants and manipulation of host gene expression, *saeR* was shown to be specifically important in phagocyte interaction. Flowing from previous observations (Rogers and Tompsett, 1952; Rogers, 1956), a model has recently been proposed, in which neutrophils have been suggested to harbour *S. aureus* and therefore act as ‘Trojan Horses’ allowing dissemination (Thwaites and Gant, 2011). The zebrafish model, therefore, provides an important platform from which to begin to determine the role of phagocytes in the dynamics of infection. This in turn may inform studies in mammalian models of *S. aureus* infection, with therapeutic relevance to human disease.

Using marked (but otherwise isogenic) strains of bacteria we have been able to identify an intraphagocyte niche for *S. aureus* host immune evasion. Mathematical modelling of the population dynamics has revealed a very small subpopulation of corrupted phagocytes, which leads to overwhelming infection. Furthermore, by applying specific phagocyte ablation strategies, we have begun to define the host cell populations responsible for *S. aureus* infection progression.

## Results

### Identification of a ‘population bottleneck’ during establishment of disseminated *S* *. aureus* bacteraemia

Following intravenous injection of 1200 cfu *S. aureus* into wild-type zebrafish larvae, bacterial numbers do not rise, and indeed often fall in the first 24 h of infection (Prajsnar *et al*., 2008). Following this, numbers increase to 10^6^ per larva with associated mortality, or bacteria are cleared in a phagocyte dependent manner. We hypothesized that *S. aureus* were avoiding host clearance by inhabiting a niche in which they could evade host immunity. To test this, and to identify the size of the niche, we generated two marked strains in an otherwise isogenic background, and co-infected zebrafish larvae with a mixture of the two in equal proportions. The SH1000 based strains JLA371 (Horsburgh *et al*., 2002) and JLA513 (Shaw *et al*., 2006) were used for these co-injections as both of them have *hla::lacZ* fusions integrated into the chromosome using pMutin-4 derivative constructs, but are importantly *hla*^+^ (Table 1). The only difference between them is the antibiotic resistance gene used for selection: JLA371 is erythromycin/lincomycin- and JLA513 tetracycline-resistant. Importantly, these strains did not differ in virulence from each other (Fig. S1A, *P* = 0.81) and the JLA371/JLA513 mixture showed similar virulence to the parental strain, SH1000 (Fig. S1B, *P* = 0.82).

**Table 1:** List of bacterial strains used in this study

Strain	Genotype or description	Reference
SH1000	Functional *rsbU*^+^ derivative of 8325-4	Horsburgh *et al*. (2002)
JLA513	SH1000 *hla*::pAISH *hla*^+^ (tet^R^)	Shaw *et al*. (2006)
JLA371	SH1000 *hla*::pMutin-4 *hla*^+^ (ery^R^)	Horsburgh *et al*. (2002)
SJF1219	SH1000 carrying pSB2035	Needham *et al*. (2004)
Newman	High level of clumping factor	Duthie and Lorenz (1952)
SJF3972	Newman *hla*::pAISH *hla*^+^ (tet^R^)	This study (transduced from JLA513)
SJF3973	Newman *hla*::pMutin-4 *hla*^+^ (ery^R^)	This study (transduced from JLA371)
SJF3665	JLA513 carrying pTKP005-YFP	This study
SJF3666	JLA371 carrying pTKP004-CFP	This study

**Fig. 1:**
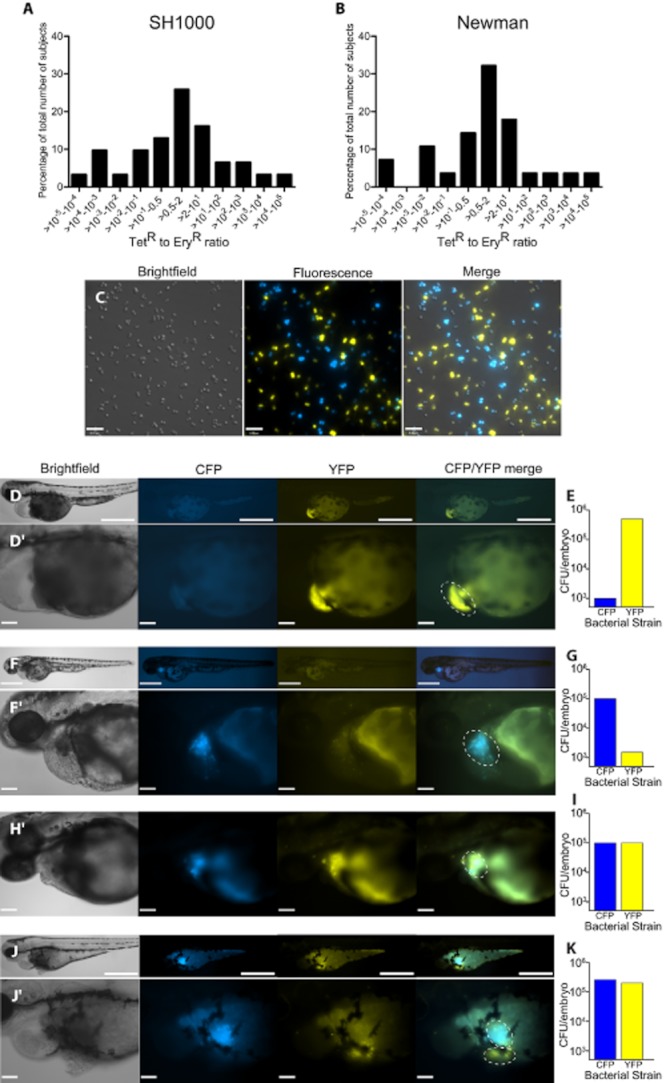
*S. aureus* subpopulations must pass a population bottleneck to establish fatal infection *in vivo*. A and B. Distribution of bacterial strain ratios (Tet^R^ to Ery^R^) recovered from terminally infected wild-type zebrafish larvae inoculated with a mixture of 600 cfu of tetracycline- and 600 cfu of erythromycin-resistant *S. aureus* SH1000 (A) or Newman (B). Larvae were homogenized at the terminal stage of infection and bacterial numbers of both strains were determined on selective media. No significant difference was observed when inter-strain ratio variances (SH1000 versus Newman) were compared using KS test (*P* = 0.64), confirming the population bottleneck effect is not strain-dependent. C. Fluorescence photomicrographs of the bacterial mixture consisting of CFP- and YFP-labelled *S. aureus* SH1000 (SJF3666 and SJF3665 respectively) prior to injection into larvae. Images were captured using 60× Nikon Plan Apo oil objective NA 1.4 and consist of a single focal plane. Scale bars represent 8 μm. D, D′, F, F′, H′, J and J′. *In vivo* images of terminally infected wild-type larvae (30–50 hpi) upon injection with a mixture consisting of 600 cfu of CFP- and 600 cfu of YFP-labelled *S. aureus* SH1000. Images were captured using 4× Nikon Plan Fluor objective NA 0.13 (D, F, J) or 10× Nikon Plan Fluor objective NA 0.30 (D′, F′, H′ and J′). Dashed ovals indicate bacterial abscess-like structures. Ten Z slices were compressed into one ‘extended focus’ image. Scale bars represent 500 μm for D, F, J and 100 μm for D′, F′, H′ and J′. E, G, I and K. Graphs representing actual bacterial burden recovered from the corresponding larvae following imaging.

Zebrafish larvae were infected with an equal co-inoculum of *S. aureus* JLA371 (Ery^R^) and JLA513 (Tet^R^) to a total of 1200 cfu. Following co-infections, larvae were maintained at 28°C and observed at intervals for the end-point of diminished blood circulation and visually identifiable bacterial lesions. Bacteria were recovered quantitatively from end-point larvae by homogenization, serial dilutions and plating on selective media to determine the total and relative bacterial numbers of each strain. The total number of bacteria at this end-point was approximately 10^6^ cfu per larva and occurred between 44 and 92 h post infection (hpi) (Fig. S1C). Bacterial population growth kinetics, during infection, matched those previously observed (Prajsnar *et al*., 2008), with a lag of 24–36 h, followed by an increase in colony-forming units, which precedes host mortality (data not shown). However, on comparison of relative numbers of each strain in the co-inoculum, in many individual larvae the output population of bacteria was asymmetrically distributed, with one or other of the original strains predominating, sometimes dramatically (Fig. 1A). Thus surprisingly, even though zebrafish larvae were infected with 600 cfu of each strain, on a large number of occasions one or other strain predominated in the final bacterial population. This suggests that not all of the inoculum was showing the same growth kinetics, otherwise all larvae would have bacterial load of approximately 50% of each type at the terminal end-point. In ecological modelling the term ‘bottleneck’ is used to describe the phenomenon whereby the final population diversity is limited by the size of the small founder population that has given rise to it. Our unexpected result suggested the presence of a ‘population bottleneck’ in the infectious process, in which the number of initial inoculum bacteria contributing to the infection end-point population is very small. To ensure this was not a strain-specific effect, the antibiotic resistance markers from JLA513 and JLA371 were transduced into the Newman strain (generating SJF3972 and SJF3973 respectively) and the co-infection experiment repeated with a similar result [Fig. 1B; Kolmogorov–Smirnoff (KS) test, *P* = 0.64].

Such unexpected overrepresentation of extreme Tet^R^ to Ery^R^ ratios could arise by the clonal expansion of an individual bacterium resulting in an end-point lesion of a single bacterial type. The transparency of the zebrafish larva enabled us to directly observe this *in vivo* using cyan fluorescent protein (CFP) and yellow fluorescent protein (YFP) (Veening *et al*., 2004) to label bacterial strains with two distinctive fluorescent tags. Vectors to enable expression of CFP and YFP were constructed based on pGL485 (Cooper *et al*., 2009) containing a constitutive *pcn* promoter under which *cfp* and *yfp* genes were cloned, producing pTKP004-CFP and pTKP005-YFP respectively (see *Experimental procedures*). Strains JLA371 (Ery^R^) and JLA513 (Tet^R^) were then transformed with pTKP004-CFP and pTKP005-YFP, resulting in SJF3666 (Ery^R^-CFP) and SJF3665 (Tet^R^-YFP) respectively. Cultures of strains SJF3666 and SJF3665 were mixed at 1:1 ratio and the resulting bacterial suspension was examined by fluorescence microscopy. Importantly, no large clumps of single labelled bacteria were seen and the bacterial strains were well mixed (Fig. 1C).

### Individual abscess-like lesions arise from individual or small numbers of bacteria

The mixed SJF3666 and SJF3665 inoculum was then used to infect London wild-type (LWT) zebrafish larvae and individuals with visible lesions on brightfield examination were imaged under fluorescence. Strikingly, each abscess-like structure seen was formed almost exclusively by bacteria with a single fluorescent label (Fig. 1D′ and F′). In about a fifth of heavily infected larvae, lesions are formed by two bacterial strains (Fig. 1H′), but in other cases they are spatially separated (Fig. 1J′). After *in vivo* imaging, larvae were homogenized and the numbers of CFP- and YFP-labelled *S. aureus* quantified within individual larvae (Fig. 1E, G, I and K), confirming the results obtained by microscopy. As the lesions are mostly populated by a single colour of fluorescent bacteria and have therefore a corresponding single drug resistance marker, they are not predominantly a mixture but are clonal. Thus, lesions are likely mostly founded by a single bacterium (or very few) as larger numbers of founders would be reflected in mixed lesions arising from the mixed inoculum.

### Favourable adaptations acquired *in vivo* do not explain the observed population distributions

To exclude confounding of these data by any intrinsic advantage of one bacterial strain over another, for example by acquisition of advantageous mutations *in vivo*, bacteria recovered from infected larvae were reintroduced into the model. Tetracycline-resistant bacteria were recovered from a larva bearing almost only tetracycline resistant organisms (hereafter called ‘Tet^R^+’ for clarity). This strain was paired with erythromycin/lincomycin-resistant bacteria recovered from the same larva recovered in very low numbers (Ery^R^−). These two isolates were co-injected in equal numbers into new sets of larvae. Co-inoculation of equal numbers of both isolates would test if the tetracycline-resistant strain had gained a selective advantage over the erythromycin/lincomycin-resistant strain. The reciprocal experiment was performed with an erythromycin/lincomycin-resistant strain recovered in high numbers (Ery^R^+) and co-injected with a tetracycline-resistant strain recovered from the same larva, but in very low numbers (Tet^R^−). No bias or selection was observed between two groups (Fig. S1D and E; Mann–Whitney test, *P* = 0.58). Thus, possible bacterial adaptation during the first infection is unlikely and the variance must result from small numbers of ‘founder’ bacteria from a privileged niche.

### Only a fraction of bacteria pass through the ‘bottleneck’ to establish disseminated infection

**Fig. 2:**
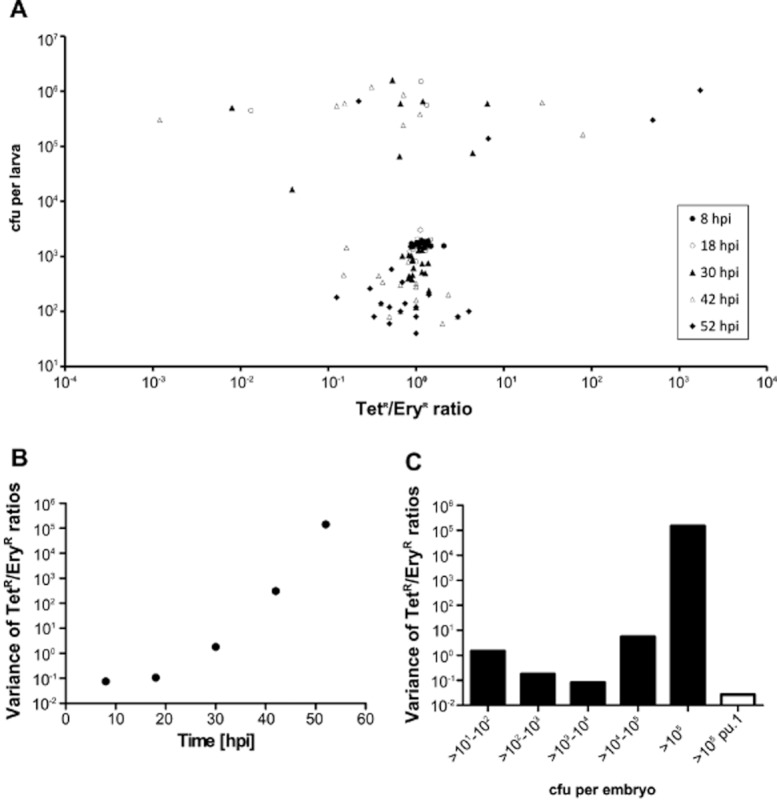
Increased bacterial ratio variance is associated with microbial proliferation *in vivo*. All graphs represent data from the same experiment. A. The relationship between total bacterial loads within larvae infected with Tet^R^/Ery^R^ mixture and bacterial Tet^R^/Ery^R^ ratio at different time points post infection. B and C. Change of Tet^R^/Ery^R^ strain variance during infection progression (B) or association with total bacterial load within infected larvae (C).

The large variance of the ratios of marked bacterial populations, combined with the observation that the abscess-like colonies are seemingly clonal, suggested that the bacteria causing disseminated infection arise from a small number of founders. However, our previous observations have shown that for the first 24–36 h, after infection with 1200 cfu, bacterial numbers in the host remain relatively constant at which point a divergence occurs where either the colony-forming units increase resulting in a terminal infection or diminish accompanied by larval survival (Prajsnar *et al*., 2008). In order to determine at which point the ratio variance increases, larvae infected with 1:1 mixture of JLA371 and JLA513 were harvested during infection progression to recover bacteria and determine the total and relative bacterial loads (Fig. 2). The increased variance in strain ratios is associated with bacterial replication and not selective death of one bacterial strain (Fig. 2A). The variance of the ratio of the two strains increases as infection progresses and is more pronounced when bacteria reach high numbers within an infected larva (Fig. 2B and C). In agreement with our previous observation (Prajsnar *et al*., 2008), bacterial numbers within an individual host do not decrease to a level consistent with a subsequent clonal expansion of the remaining bacteria leading to terminal infection. In addition, if bacterial numbers decrease to approximately 100 within a host, no skewing of strain ratio was observed.

### The ‘population bottleneck’ is dependent on phagocyte function

**Fig. 3:**
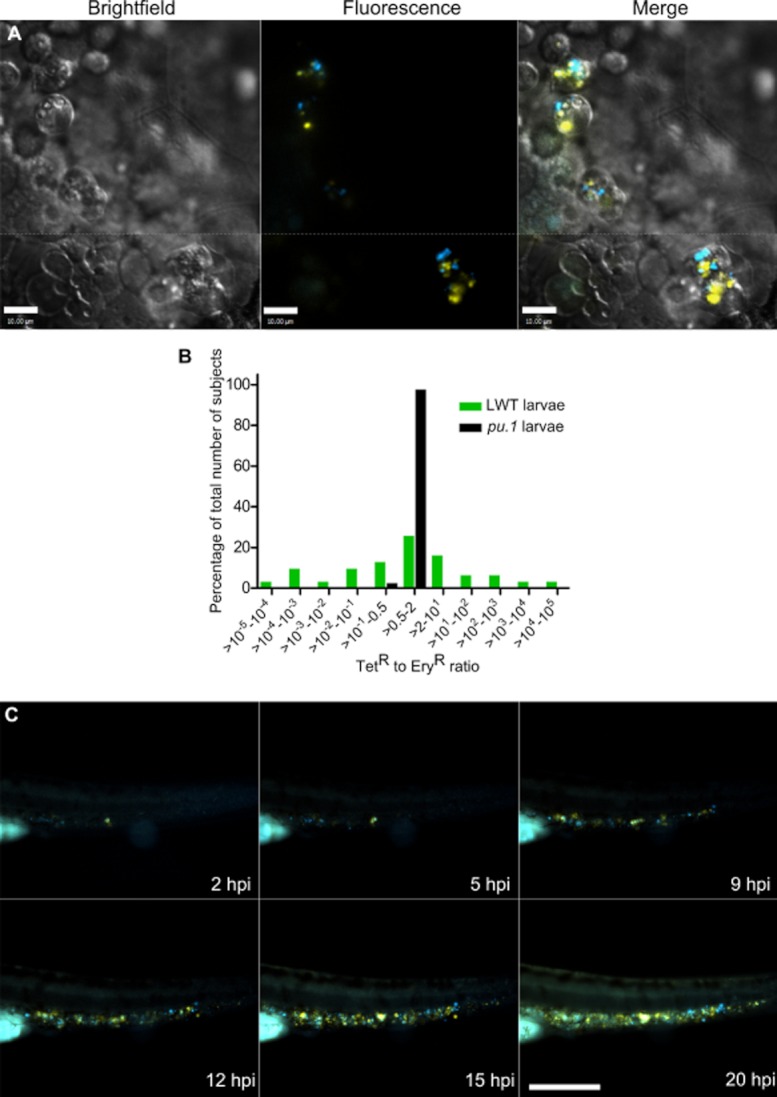
Phagocytes are required for the population bottleneck. A. *In vivo* images of the yolk circulation valley of 32 hpf larvae, 2 h after injection with a mixture consisting of 600 cfu of CFP- and 600 cfu of YFP-labelled *S. aureus*. Two bacterial strains were equally distributed within zebrafish phagocytes. A dashed horizontal line separates two parts of the image captured at different focal planes. Scale bars represent 10 μm. B. Variance of bacterial strain (Tet^R^ to Ery^R^) ratio from *S. aureus* infected phagocyte-depleted zebrafish larvae (wild-type larvae from Fig. 1A were also shown for comparison). Larvae were homogenized at the terminal stage of infection and bacterial numbers of both strains were determined on selective media. KS test analysis showed that the difference in the variance of bacteria ratios between *pu.1* and LWT is highly significant (*P* < 0.001). C. Microscopy images from a time-lapse series of larvae infected with *S. aureus* by injection with a mixture consisting of 600 cfu of CFP- and 600 cfu of YFP-labelled *S. aureus* into the bloodstream of a *pu.1* knock-down larva. Images were captured at the time intervals indicated, using 10× Nikon Plan Fluor objective NA 0.13 and consist of a single focal plane. Scale bar represents 200 μm. The full time-lapse data set from which this figure is taken can be seen as Video S1.

During the *in vivo* imaging of larvae infected with CFP- and YFP-labelled *S. aureus* strains by 2 hpi, bacteria are found within phagocytes (Fig. 3A). Imaging confirmed the two bacterial strains were equally distributed within phagocytes, and some phagocytes only contain small (up to five) numbers of bacteria. We have previously shown that phagocytes are necessary for host resistance to *S. aureus* infection in the zebrafish by preventing early uncontrolled proliferation in the circulation (Prajsnar *et al*., 2008). We therefore examined the effect on the population distribution of phagocyte ablation using a morpholino against *pu.1*, a transcription factor essential for development of myeloid cells (Rhodes *et al*., 2005). Almost all larvae showed signs of end-point infection within 26 h, and bacteria recovered from these larvae reached approximately 10^6^ cfu per larva. Interestingly, the variance of bacterial ratios recovered from these larvae was extremely small. In fact, only about 2% (one out of 40 larvae) bore one of the two strains at more than twofold higher number than the other (Fig. 3B). By comparison, in wild-type larvae the overall strain distribution was heavily skewed in favour of one or the other of the strains (ranging from 80 000:1 to 1:15 000). The KS test for comparison of variance showed that the difference in the bacterial ratio variances between *pu.1* and LWT is highly significant (*P* < 0.001). A clear demonstration of this was seen when *pu.1* knock-down larvae were injected with a mixture of CFP- and YFP-labelled *S. aureus* and the infection progression was followed using fluorescence microscopy. Low bacterial ratio variance was observed, as both CFP- and YFP-labelled strains proliferated at the same rapid rate (Fig. 3C, Video S1). It is important to note that although bacterial numbers reached 10^6^ cfu at terminal stages of *pu.1* larvae infection, strain ratio variance remained very low (see Fig. 2C for comparison) providing additional evidence that high variance is not simply a consequence of high bacterial loads.

We conclude that host phagocytes are absolutely required for the observed bottleneck in infection, leading us to term this an ‘immunological bottleneck’ since it is defined by the action of the innate immune system. The *pu.1* knock-down data also suggest that the bottleneck occurs within phagocytes and that a small subpopulation of bacteria is protected within this niche, which then subsequently escapes to cause an overwhelming infection.

### A mathematical model of disease progression is consistent with the hypothesis that a few phagocytes are responsible for the overwhelming infection

Mathematical modelling of population dynamics has led to major advances in understanding many processes, including bacterial infection (Levin *et al*., 1999). To better understand the origins of the variance of bacterial populations observed, a mechanistic mathematical model of pathogen population dynamics during infection was developed and challenged with the experimental data. Our first model looked at the distribution of ratios that we would expect in a sample of infected larva under the assumption that, at death, each bacterium has a 50:50 chance of being one strain or the other. This resulted in a distribution of ratios with a median value of 1.00 and variance of 2 × 10^−9^ compared to the observed data which has a median value of 1.04 but a variance, at 1.4 × 10^5^, many orders of magnitude larger, and therefore clearly shows that another process is needed to explain the extreme variance in our data.

**Fig. 4:**
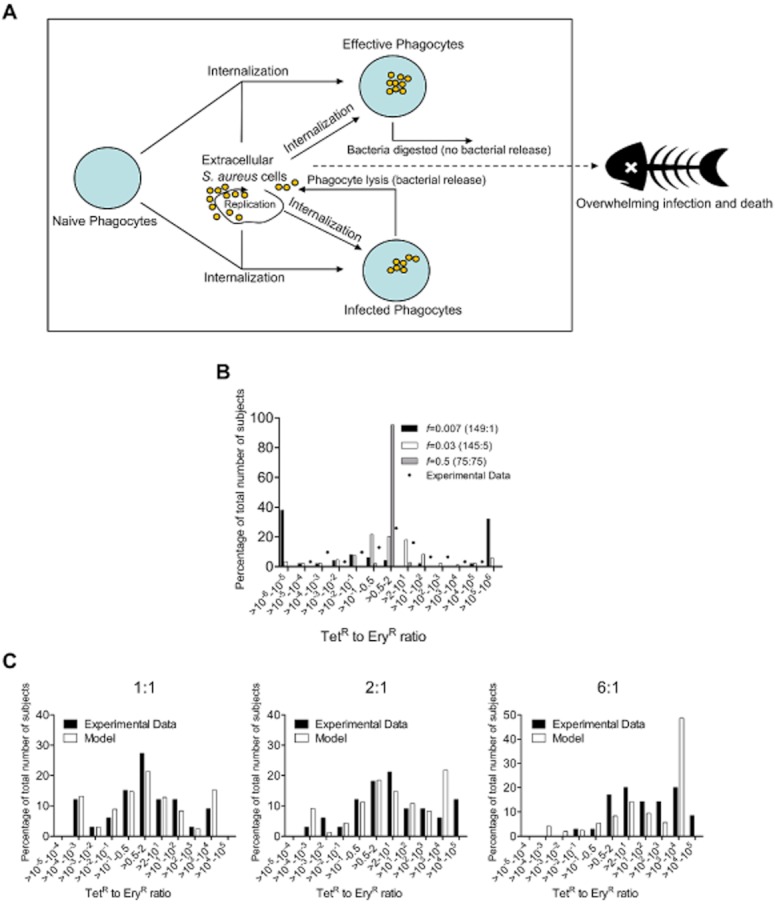
Population modelling demonstrates the immunological bottleneck is comprised of a small number of phagocyte niches. A. A schematic diagram representing the key variables and processes included in the model. Naïve phagocytes, on encountering a bacterial cell, can internalize them through normal processes and become ‘Effective’ phagocytes, leading to the control of the bacteria. Or, with a small probability, *f*, this process is subverted and the phagocyte becomes an ‘Infected’ phagocyte, allowing the eventual release of viable bacteria. B. Effect of varying probability of phagocyte subversion, *f*, on Tet^R^ to Ery^R^ ratio at time of death for 1000 simulations of the model. For comparison, the experimental data is included. Numbers in brackets represent hypothetical number ratios of effective to infected phagocytes for a given *f*. C. Comparison of 1000 simulations of the model to experimental data of Tet^R^ to Ery^R^ ratios at time of death for three different initial dose ratios (1:1, 2:1 and 6:1 Tet^R^ to Ery^R^ respectively) (*f* = *0.03*).

We then aimed to establish whether an intraphagocyte niche was compatible with the observed data. To do this, we developed a within-host infection model that captures the interaction between the bacteria and the general phagocyte population. The model considers a host as consisting of a population of freely mobile phagocytes (approximately 150 cells, although the model is relatively insensitive to the value of this parameter), which is then inoculated with a small population (dose) of *S. aureus*. The key assumption of the model is that in a small fraction of phagocytes (which we term ‘infected’ phagocytes) internalized bacteria subvert the normal processes of bacterial killing, resulting in release of viable bacteria. We further assume there is no replication of bacteria within phagocytes, but that phagocytes can continue to internalize bacteria while they remain viable. Previously, it has been suggested that *S. aureus* in able to survive without replication in neutrophils, but ultimately cause lysis (Voyich *et al*., 2005; Kobayashi *et al*., 2010). In our proposed model, as in our observations, terminal infection occurs when the population of *S. aureus* within the host reaches a threshold value (Fig. 4A).

The model was put into an individual based model framework to allow us to capture the stochastic nature of the underlying processes. We initiated the model with a 1:1 ratio of the two bacterial strains and the population dynamics of the extracellular bacteria and the two subpopulations of phagocytes were then tracked. Using an individual-based model framework allowed us to follow the specific fate of the different bacterial strains. The *S. aureus* growth model (Fig. 4 and Table S1) was generated, and the results (after 1000 iterations) showed a very small number of infected phagocytes – around 3% – within an infected larva can give rise to the extreme variance ratios we see in the experimental data (Fig. 4B).

We tested the model by fixing the probability of infected phagocyte production at 3%, (*f* = 0.03) and used the model to predict the outcome when the inoculant dose was skewed. Using various inoculant ratios we obtained the ratio of the two strains at time of death for 1000 simulations where the outcome was death rather than resolution of infection. After assessing the model output, inoculant dose ratios of 1:1, 1:2 and 1:6 Tet^R^/Ery^R^ were selected to test experimentally, because the model suggested an identifiable trend with these doses would be observed. Figure 4C shows the comparison between 1000 simulations of the model at each dose ratio and the experimental results obtained using each inoculant dose. At the 1:1 dose ratio, a clearly comparable pattern between the model prediction and the results obtained experimentally is seen, with a little over 20% of cases resulting in extreme Tet^R^ to Ery^R^ ratios (either very high or very low). Additionally, both the model and the experimental data show peaks where the Tet^R^ to Ery^R^ ratio is approximately equivalent in around 20–30% of cases. With an inoculant dose ratio of 1:2, the model predicts that there will be more cases where the variance is biased towards the strain that was greater in the initial dose. Crucially, however, it also predicts that there will still be occasional cases where the strain that was reduced in the dose is dominant in ratio at time of death. These predictions are borne out by the experimental data (Fig. 4C middle). A similar, but more exaggerated pattern is predicted for the case where the inoculant dose is 1:6, with the model predicting around 50% of cases where the initially dominant strain is found at levels around 10 000–100 000 times higher than the lesser strain. There do however, remain a small number of cases where the initially smaller strain is found in higher numbers than the strain with the initially higher dose: a notable feature of both the model, and the experimental data. Overall our model is consistent with the hypothesis that a small number (around 3%) of infected phagocytes serve as an intracellular reservoir for *S. aureus*, and upon their lysis subsequently lead to disseminated infection, as has been suggested in the mammalian system (Voyich *et al*., 2005; Kobayashi *et al*., 2010).

### Genetic manipulation of specific phagocyte numbers suggests neutrophils are the intracellular reservoir for *S. aureus*

**Fig. 5:**
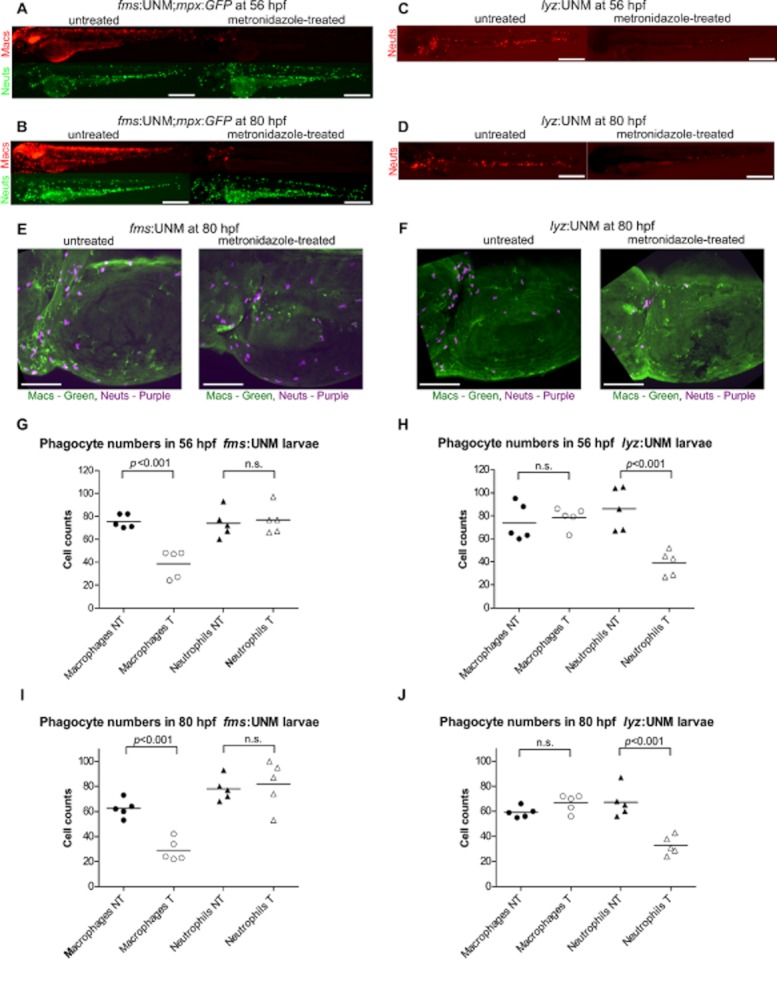
Metronidazole treatment of *fms*:UNM and *lyz*:UNM causes effective depletion of macrophages and neutrophils respectively. A–D. *In vivo* images of *fms*:UNM;mpx:*GFP* larvae at 56 hpf (A) and 80 hpf (B); and *lyz*:UNM larvae at 56 hpf (C) and 80 hpf (D) non-treated and treated overnight (34–50 hpf) with 6.5 mM metronidazole. In A and B, macrophages (Macs) appear as red foci (due to mCherry), whereas neutrophils (Neuts) are shown in green (due to GFP). In C and D, neutrophils (Neuts) appear as red foci (due to mCherry). Images were captured using 2× Nikon Plan UW objective NA 0.06. Ten Z slices were compressed into one ‘extended focus’ image. Scale bars represent 500 μm. E and F. Microscopic images of *fms*:UNM (E) and *lyz*:UNM (F) larvae subjected to Cy5-TSA and anti-L-plastin whole mount staining. Macrophages (Macs) appear green (Alexa Fluor 488), whereas neutrophils (Neuts) are shown in purple (Cy5-TSA). Images were taken using 10× Olympus UPlanSApo objective NA 0.40 and each consist of a single focal plane. Scale bars represent 200 μm. G–J. Phagocyte (neutrophil and macrophage) counts in *fms*:UNM (G – 56 hpf, I – 80 hpf) and *lyz*:UNM (H – 56 hpf, J – 80 hpf) larvae non-treated (NT) or following overnight (34–50 hpf) treatment with 6.5 mM metronidazole (T). Each point indicates a sum of counts from yolk sac and trunk area of a single larva. Significant differences in cell numbers are indicated.

In order to determine whether neutrophils and/or macrophages are the site of the immunological bottleneck, the ability to specifically ablate each cell types was exploited. The nitroreductase gene (*nfsB*) converts the pro-drug metronidazole to a cytotoxic metabolite, and has been used to successfully ablate cell populations in the zebrafish (Pisharath *et al*., 2007). A transgenic line expressing *nfsB*.mCherry under the upstream activator sequence (UAS) promoter was crossed to macrophage (*fms*:Gal4) (Gray *et al*., 2011) and neutrophil (*lyz:Gal4*) promoter driven Gal4 lines (Elks *et al*., 2011). Thirty-four hours post fertilization (hpf), transgenic *fms*:UNM (Gray *et al*., 2011) or *lyz*:UNM (Elks *et al*., 2011) larvae were subjected to overnight metronidazole treatment allowing partial elimination of macrophage and neutrophils respectively (Fig. 5). The cell ablation efficacy was assessed by live fluorescence microscopy (Fig. 5A–D) as well as a combination of histochemical staining for endogenous peroxidase activity (*mpx*, expressed in neutrophils) and L-plastin immunostaining (present in both leukocyte types) (Fig. 5E–J). Approximately 50–55% ablation efficacy (in yolk sac and trunk, from ∼ 80 phagocyte cells in untreated to ∼ 40 in treated larvae) could be achieved and specific ablations were maintained for at least another 24 h (Fig. 5G–J), with similar efficacy. For macrophage ablation, the number of neutrophils remained unaffected and *vice versa* (Fig. 5G–J).

**Fig. 6:**
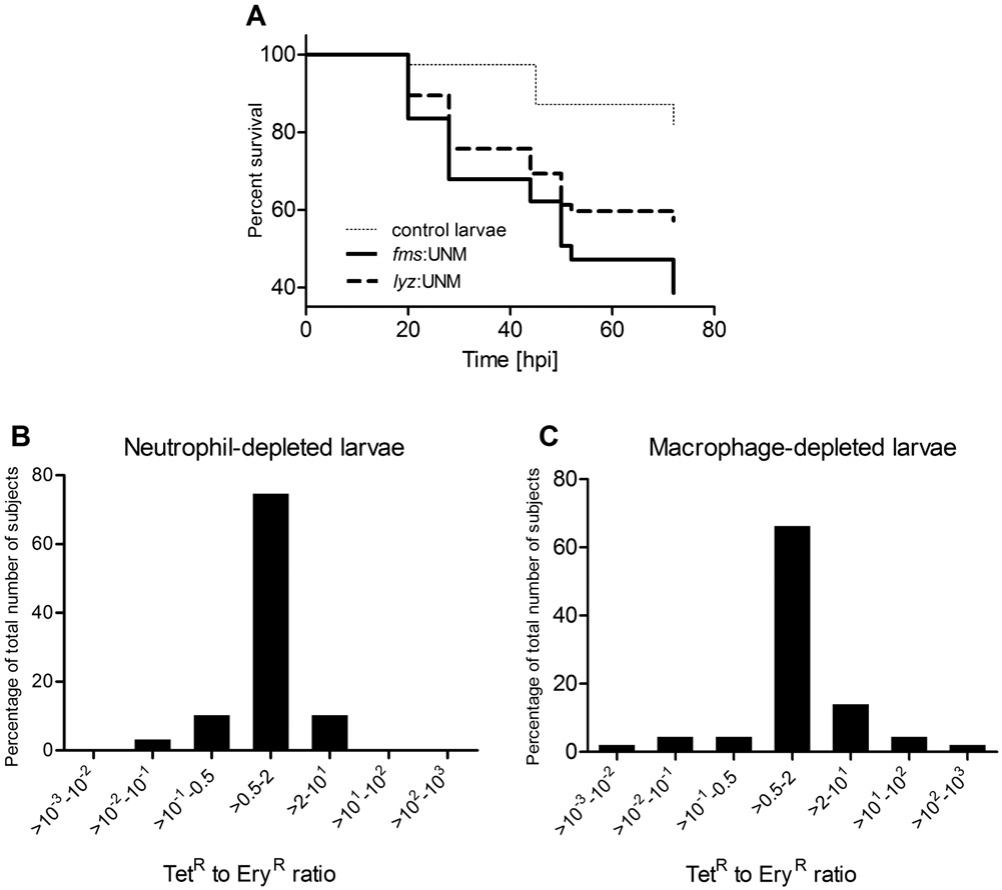
Both macrophages and neutrophils are required for immunity against *S. aureus*, but neutrophils may also provide an intracellular niche for bacteria. A. Survival of macrophage- (*fms*:UNM) or neutrophil-depleted (*lyz*:UNM) and wild-type larvae upon injection with 4000 cfu of *S. aureus* SH1000 into the circulation (*n* ≥ 40). Pairwise comparisons: *P* < 0.0001 for *fms*:UNM versus wild-type larvae, *P* = 0.005 for *lyz*:UNM versus wild-type larvae and *P* = 0.005 for *fms*:UNM versus *lyz*:UNM. B and C. Distribution of bacterial strain ratios (Tet^R^ to Ery^R^) of JLA513 and JLA371 in infected neutrophil- (B) and macrophage-depleted (C) zebrafish larvae. Larvae were homogenized at the terminal stage of infection and bacterial numbers of both strains were determined on selective media. The KS test analysis shows that the difference in the variance of bacteria ratios between neutrophil- and macrophage-depleted larvae is significant (*P* = 0.015).

After metronidazole treatment, 54 hpf larvae ablated of either macrophages or neutrophils were then challenged with 4000 cfu of *S. aureus* SH1000 to determine the importance of particular phagocyte type in host immunity to staphylococcal infection. A higher bacterial dose was used, as control, 54 hpf larvae showed notably greater resistance to *S. aureus* (likely due to higher phagocyte number) in comparison to 30 hpf larvae. Both macrophages and neutrophils play a role in preventing infection, as ablated groups were significantly more susceptible to *S. aureus* compared with controls (Fig. 6A, *P* ≤ 0.005). However, loss of macrophages had more detrimental effect in comparison to neutrophil depletion (*P* = 0.005), in keeping with the increased ability of zebrafish macrophages to effectively phagocytose bacteria in suspension (Colucci-Guyon *et al*., 2011).

In order to determine which phagocyte type might be responsible for the immunological bottleneck observed during infection, both macrophage- and neutrophil-ablated larvae were injected with a mixture of Tet/Ery-resistant strains. Neutrophil-depleted larvae had significantly reduced strain ratio variance (Fig. 6B) compared to macrophage-depleted counterparts (Fig. 6C, KS test, *P* = 0.015), suggesting that neutrophils may be the immunological bottleneck during *S. aureus* infection.

**Fig. 7:**
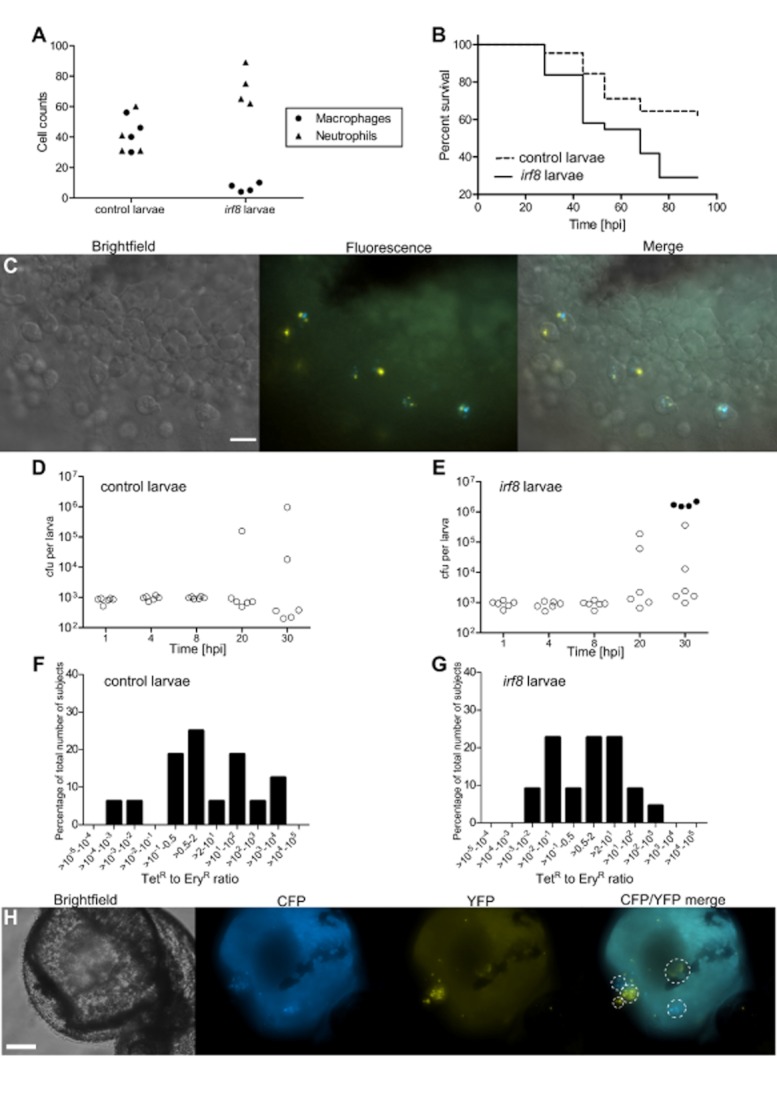
Increasing neutrophil numbers with an *irf8* morpholino alters *S. aureus* population dynamics. A. Phagocyte (neutrophil and macrophage) counts of the yolk sac area in wild-type and *irf8* knock-down larvae. In order to visualize neutrophils and macrophages, larvae were subjected to Cy5-TSA and anti-L-plastin whole mount staining. B. Survival of wild-type and *irf8* knock-down larvae after injection with 1000 cfu of *S. aureus* SH1000 into the circulation (*n* = 20). *P* = 0.0037 for *irf8* knock-down versus wild-type larvae. C. *In vivo* images of the yolk sac circulation valley of 32 hpf *irf8* knock-down larvae 1 h after injection with a mixture consisting of 600 cfu of CFP- and 600 cfu of YFP-labelled *S. aureus* SH1000. Images were captured using 6× Nikon Plan Apo oil objective NA 1.4 and consist of a single focal plane. Scale bars represent 10 μm. D and E. Growth of *S. aureus* SH1000 within wild-type (D) and *irf8* knock-down (E) larvae after injection with 1000 cfu of *S. aureus* SH1000 into the circulation. At each time point a group of six living larvae (open circles) plus any dead larvae (filled circles) were collected and bacterial numbers (cfu per larva) determined. F and G. Distribution of bacterial strain ratios (Tet^R^ to Ery^R^) of JLA513 and JLA371 in infected wild-type (F) and *irf8* knock-down (G) zebrafish larvae. Larvae were homogenized at the terminal stage of infection and bacterial numbers of both strains were determined on selective media. The KS test showed that this difference in the variance of bacteria ratios between wild-type and *irf8* knock-down larvae is not significant (*P* = 0.53). H. *In vivo* images of *irf8* knock-down larvae 24 h upon injection with a mixture consisting of 600 cfu of CFP- and 600 cfu of YFP-labelled *S. aureus* SH1000. Images were captured using 10× Nikon Plan Fluor objective NA 0.30. Multiple foci of infection which are likely to originate from a single bacterial cell are indicated with dashed ovals. Ten Z slices were compressed into one ‘extended focus’ image. A scale bar represents 100 μm.

If neutrophils are the site of the niche from which *S. aureus* founds the fatal infection, then it might be expected that increasing the number of neutrophils would lead to an increase in dissemination. Therefore, neutrophil numbers were increased by a genetic strategy. In zebrafish larvae, myelopoiesis can be reorientated by modulating levels of interferon regulatory factor-8 (*irf8*), which is required for differentiation of myeloid progenitor cells into macrophages (Li *et al*., 2011). Therefore, in order to increase zebrafish neutrophils (at the expense of macrophages) *irf8* knock-down larvae were generated by injecting *irf8* MO^atg^ (Li *et al*., 2011). To confirm efficacy, numbers of neutrophils and macrophages were counted at 32 hpf and compared to controls. Numbers of neutrophils (in the yolk sac area) were 41 ± 7 in controls versus 73 ± 6 in *irf8* larvae, whereas numbers of macrophages were 43 ± 5 in controls versus 7 ± 1 in *irf8* larvae, suggesting a strong shift in myelopoiesis towards neutrophil formation in *irf8* knock-down larvae (Fig. 7A). As expected, these larvae were hypersusceptibile to *S. aureus* infection (Fig. 7B), but retained the ability to phagocytose bacteria normally. Phagocytosis efficacy was assessed by *in vivo* microscopic imaging of *irf8* knock-down larvae 2 hpi a mixture consisting of 600 cfu of CFP- and 600 cfu of YFP-labelled *S. aureus* SH1000. By 1 h following injection, all visible bacteria were contained within phagocytes (Fig. 7C). Imaging of *irf8* knock-down larvae was supported by quantitating bacterial population kinetics *in vivo* for time intervals up to 30 hpi. Bacterial numbers did not increase significantly in the first 8 h after infection suggesting that all bacteria have been phagocytosed (Fig. 7D and E). This is in contrast to *pu.1* morphants, in which bacteria grew exponentially from the time of infection (Prajsnar *et al*., 2008).

In order to determine if, in neutrophil-enriched larvae, the capacity of the immunological bottleneck is increased during infection, *irf8* knock-down larvae were injected with a mixture of Tet/Ery-resistant strains. No significant difference was observed between *irf8* morphant and control larvae (Fig. 7F and G; KS test, *P* = 0.53). However, this experiment could be confounded by the fact that in the neutrophil-enriched *irf8* morphant larvae, more individual foci of infection might occur. Although each of these foci might be founded by a single bacterium, collectively the variance of strain ratios might remain low: the collective behaviour masking the behaviour in individual foci of infection. Therefore, the experiment was repeated using the fluorescent marked strains to test whether *irf8* knock-down zebrafish larvae inoculated with the mixed inoculum of CFP and YFP strains had more abscess-like structures. *In vivo* imaging revealed that as infection progresses, more lesions are formed in *irf8* knock-down larvae than those observed in their wild-type siblings (Figs 7H and S2). Multiple lesions originating from different bacterial strains were seen within *irf8* knock-down larvae, suggesting the formation of independent abscesses in greater numbers than seen in wild-type counterparts.

### In a systemic mammalian model of infection, abscesses are founded by individual bacteria

**Fig. 8:**
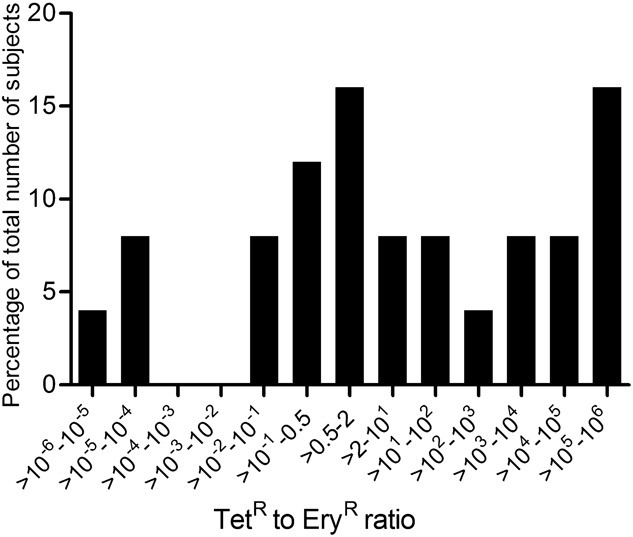
High strain ratio variance is also seen in a mouse systemic model of *S. aureus* infection. Distribution of bacterial strain ratios recovered from kidneys harvested from mice after infection with a mixture of 5 × 10^6^ cfu of tetracycline- and 5 × 10^6^ cfu of erythromycin-resistant *S. aureus* SH1000. After 10 dpi, mice were sacrificed and kidneys aseptically removed and bacteria enumerated.

The zebrafish is a useful model of *S. aureus* infection. However, its importance to human disease can only be verified if findings can be translated to mammalian systems. In order to determine if the immunological bottleneck is a general phenomenon of *S. aureus* infection, we used the well-established septic arthritis mouse model of staphylococcal infection (Tarkowski *et al*., 2001). In this system, bacteria are injected into the tail vein and subsequently abscesses are formed in the kidneys. Mice were infected with an equal co-inoculum of *S. aureus* JLA371 (Ery^R^) and JLA513 (Tet^R^) to a total of 1 × 10^7^ cfu. For those kidneys that showed external abscesses (usually one or two), individual abscesses were excised and bacterial levels enumerated, alongside the rest of the remaining kidney material. For all other kidneys the entire kidney was homogenized. Figure 8 shows the data gathered from whole kidneys after infection (including external abscesses recovered from particular kidneys) demonstrating a large variance, with many kidneys containing almost exclusively a single strain type. Again this variance is much higher than would be expected by chance. A null model, assuming each bacterium has a 50% chance of being either strain, would predict a variance of ratios of 3.3 × 10^−8^ compared to the observed variance, which is many orders of magnitude larger (5.8 × 10^11^). Table S2 shows individual abscess data where out of 15 recovered abscesses, only two contained both Tet^R^ and Ery^R^ strains (< 99:1 of one strain versus the other). This suggests that murine kidney abscesses, similar to the comparable lesions in zebrafish larvae, are usually founded by a single bacterium.

## Discussion

Several techniques have been used to study pathogen dynamics *in vivo*, including bioluminescent imaging using luciferase-labelled bacteria (Xiong *et al*., 2005). Such non-invasive methods are not sensitive enough to offer the resolution required for quantitative modelling. On the other hand, although laborious, experimental infections involving multiple marked strains have proven to be of great potential for understanding mechanisms of pathogenesis (Moxon and Murphy, 1978; Brown *et al*., 2006; Margolis and Levin, 2007; Grant *et al*., 2008). Here, we used marked strains in an established zebrafish model of systemic *S. aureus* infection to reveal an immunological bottleneck, which results in terminal infection initiated by just one or two individual bacteria from the initial inoculum.

Our data suggest that *S. aureus* requires a high inoculum in many models to initiate infection because there are limited host niches where bacteria can evade host killing and subsequently initiate fatal infection. Additionally, in the zebrafish, although few *S. aureus* cells initiate terminal disease, other bacteria are not promptly killed following phagocytosis, but contained within phagocytic cells, that prevent rapid bacterial proliferation. The hypothesis postulated by Schmid-Hempel and Frank that higher inocula of *S. aureus* are needed in order to establish threshold concentrations of secreted virulence factors (Schmid-Hempel and Frank, 2007) does not explain the high bacterial ratio variance observed in our model.

The variance observed cannot be explained by selection of mutants from within the bacterial population, as ‘selected’ bacterial clones isolated from terminally infected subjects did not show enhanced virulence in subsequent infection experiments. Conversely, in a study of rat nasal colonization by *Haemophilus influenzae* and subsequent systemic infection, it was hypothesized that in some cases bacteria are subject to within-host evolution and therefore some subsets could become more invasive than others (Margolis and Levin, 2007).

Depletion experiments clearly demonstrated the importance of phagocytes in generating an immunological bottleneck during infection, as loss of phagocytes in the *pu.1* morphant resulted in complete loss of variance. Similarly, in a study by Grant *et al*. using tagged, but otherwise isogenic strains of *Salmonella enterica* to determine the within-host bacterial dynamics in mice, phagocytes were found to be responsible for individual strain predominance (Grant *et al*., 2008).

Therefore, there is an immunological bottleneck in the zebrafish model of *S. aureus* bacteraemia, which explains the need for a high inoculum to cause overwhelming infection. This suggests there are very few effective niches within a host and a high inoculum is required in order to fill them and cause later disseminated infection. The niches are a feature of the host rather than the pathogen: there is no favoured subpopulation of bacteria. A mathematical model was developed in order to predict the number of niches (infected phagocytes), allowing *S. aureus* cells to pass the immunological bottleneck. The experimental data show broad agreement with the model predictions across a wide range of conditions. However, in certain cases, the model predicts more extreme behaviour than is seen in the data. There are several reasons that might account for this: we have constructed an extremely parsimonious model, limiting the interactions to those between phagocytes and bacteria as this appears to capture the essential dynamics. However, there are numerous other processes occurring simultaneously that may be affecting the system – other host immune responses and resource availability to both the host and bacteria (Goelzer and Fromion, 2011) being perhaps the most significant. Experimental constraints also mean that sample sizes are necessarily small compared to those obtainable from model simulations.

We have used a very simple model that clearly suggests that a single, straightforward mechanism can give rise to the notable experimental data. Given biologically identical bacterial strains, the extreme strain ratios at time of death can arise if there is a small probability that a biologically functional phagocyte can be subverted upon infection by *S. aureus*, such that it is no longer able to sequester its bacterial contents but instead releases viable bacteria back into the host. In a host with 150 phagocyte cells, the number of affected cells would be in the order of five.

A key question then arises as to what is the nature of the phagocyte that leads to founding of a terminal infection. Phagocyte type specific ablations were performed to determine if one cell type confers host defence against *S. aureus*, with the other acting as a potential pathogen reservoir. The ablations performed were incomplete, most likely due to inactivation of the 14× UAS by methylation (Goll *et al*., 2009). It has been previously shown that although both macrophages and neutrophils can phagocytose invading pathogens within zebrafish larvae; macrophages ingest bacteria with greater efficiency (Le Guyader *et al*., 2008; Prajsnar *et al*., 2008). Here, both cell types are important in preventing overwhelming staphylococcal infection in zebrafish larvae, which concurs with mammalian systems where ablation of either neutrophils or macrophages was deleterious to the host upon systemic *S. aureus* infection (Verdrengh and Tarkowski, 1997; 2000). Additionally, here we show that even partial loss of neutrophils results in reduced bacterial ratio variance, suggesting that indeed neutrophils may form a reservoir during the infection. In addition, enhanced mortality and increased numbers of foci of infection in neutrophil-enriched (*irf8* knock-down) infected larvae, also suggesting that neutrophils form the immunological checkpoint for progression to an overwhelming infection. Interestingly, the number of foci of infection increases associated with the rise in neutrophil levels upon *irf8* knock-down, further suggesting that neutrophils carrying *S. aureus* cells indeed could be the ‘Trojan Horses’ (Thwaites and Gant, 2011) facilitating overwhelming infection.

It has been demonstrated that a partial depletion of murine neutrophils using monoclonal antibodies increases mouse survival when challenged intraperitoneally with *S. aureus* (Gresham *et al*., 2000). This suggests that neutrophils, in addition to their beneficial role in bacterial clearance, may provide an intracellular reservoir for persisting bacteria. Similar observations have been made in a zebrafish larval model of *Mycobacterium marinum*, where bacteria, injected into the contained space of the hindbrain ventricle, are disseminated throughout the body inside macrophages (Clay *et al*., 2007). In phagocyte-depleted larvae, infecting mycobacteria fail to disseminate to tissues. Neutropenic patients are highly susceptible to *S. aureus* infections (Hersh *et al*., 1965; Rubio *et al*., 1994). However, our data and that of others suggest that neutrophils are also responsible for disease progression and dissemination (Gresham *et al*., 2000; Voyich *et al*., 2005; Kobayashi *et al*., 2010).

For many years *S. aureus* was considered as an extracellular pathogen. However, many recent reports have shown the ability of *S. aureus* to survive in human cells, including professional phagocytes such as neutrophils (Gresham *et al*., 2000) and macrophages (Kubica *et al*., 2008), subsequently leading to cell lysis and bacterial release. It has been demonstrated that *S. aureus*-infected neutrophils transferred from a diseased into a naïve mouse can establish subsequent infection (Gresham *et al*., 2000). These observations have led to the hypothesis that the mode of entry into neutrophils might define the outcome of that interaction (Gresham *et al*., 2000). It has been suggested that wild-type *S. aureus* can be internalized by murine neutrophils by two mechanisms: phagocytosis and macropinocytosis. Zipper-like phagocytosis leads to formation of tight phagosomes containing bacteria, which are competent for fusion with azurophil granules, allowing subsequent bacterial destruction. The alternative process, macropinocytosis, results in bacteria being taken up into large spacious macropinosomes, which may not be competent for fusion which granules and therefore permit *S. aureus* to lyse the vacuolar membrane and escape into the cytoplasm (Gresham *et al*., 2000). Macropinocytosis has been observed in zebrafish kidney phagocytes (monocytes/macrophages and neutrophils) and this process is utilized in *Edwardsiella ictaluri* infection (Hohn *et al*., 2009). This suggests a potential mechanism for *S. aureus* survival and subsequent escape from zebrafish neutrophils.

The zebrafish data here informed the use of a mammalian systemic model of infection. Mice injected with mixed bacterial strains led to foci of infection (kidney abscesses) likely initiated by single bacteria. Previously, using two capsule variant strains of *S. aureus*, Watts *et al*. found infected kidneys containing single clones (Watts *et al*., 2005). Furthermore, in our study we have also observed several kidneys with more than one abscess (each originating from a single bacterium), but also kidneys containing both types of marked strains, suggesting independent formation of abscesses within the same kidney.

How do so few *S. aureus* avoid phagocyte killing within mammalian and zebrafish hosts? It would seem these bacteria generate a protective environment within a phagocyte, from which they may initiate foci of infection that ultimately become abscesses. This highlights the possibility of targeting intracellular bacteria as a way to reduce initiation of abscess formation and so lessen the bacterial load with important consequences for patient well-being.

## Experimental procedures

### Ethics statement

All animal work was performed according to guidelines and legislation set out in UK law in the Animals (Scientific Procedures) Act 1986. Ethical approval was given by the University of Sheffield Local Ethical Review Panel.

### Bacterial strains and growth conditions

*Staphylococcus aureus* strains (listed in Table 1) were grown in brain heart infusion (BHI) broth medium (Oxoid) at 37°C supplemented with antibiotics where appropriate at the following concentrations: chloramphenicol 30 μg ml^−1^, tetracycline 5 μg ml^−1^, erythromycin 5 μg ml^−1^ and lincomycin 25 μg ml^−1^.

### Construction of bacterial fluorescence reporters

Genes encoding CFP and YFP were PCR-amplified from plasmids pICFP and pIYFP respectively (Veening *et al*., 2004) using the following primer pair (forward: ATAATAGGGCCCATAAAGGAGGATCGAATTCTTGGATTC, reverse: ATAATAGGATCCTTACTTGTACAGCTCGTC). The PCR products were then cloned into pGL485 (Cooper *et al*., 2009) under the control of the constitutive *pcn* promoter, in order to provide stable expression. Inserts and the plasmid were restriction digested using ApaI and BamHI prior to ligation. The resulting plasmids, pTKP003-CFP and pTKP004-YFP, were introduced into *S. aureus* RN4220 by electroporation, resulting in transformants expressing *cfp* and *yfp* (verified by fluorescence microscopy) The plasmids were then transferred into *S. aureus* SH1000 by Φ11 transduction, and transductants verified by fluorescence microscopy.

### Zebrafish strains and maintenance

London wild-type, *Tg(fms*:*GAL4);Tg(UAS:nfsB.mCherry)* and *Tg(fms*:*GAL4);Tg(UAS:nfsB.mCherry);Tg(mpx*:*GFP)* fish (hereafter referred to as LWT, *fms*:UNM or *fms*:UNM;*mpx*:*GFP* respectively for clarity) were used and have been previously described (Renshaw *et al*., 2006; Gray *et al*., 2011). The *Tg(lyz*:*GAL4)* line generated previously (Elks *et al*., 2011) was crossed with *Tg(UAS:nfsB.mCherry)* fish (Gray *et al*., 2011) and the offspring expressing mCherry were raised to adulthood to produce a stable *Tg(lyz:GAL4);Tg(UAS:nfsB.mCherry)* line (thereafter referred to as *lyz*:UNM for clarity). Larvae were incubated in E3 medium at 28°C according to standard protocols (Nusslein-Volhard and Dahm, 2002).

### Morpholino knock-down of *pu.1* and *irf8*

Morpholino-modified antisense oligomers against *pu.1* (Rhodes *et al*., 2005) and *irf8* (MO^atg^) (Li *et al*., 2011) were injected using a method described previously (Prajsnar *et al*., 2008). A standard control morpholino (Genetools) was used as a negative control.

### Specific macrophage/neutrophil depletion

Specific macrophage depletion was achieved by incubation of 34 hpf *fms*:UNM or *fms*:UNM;*mpx*:*GFP* larvae in 6.5 mM of metronidazole dissolved in E3 medium for 18 h. The same method was performed to achieve neutrophil depletion using *lyz*:UNM larvae. AB larvae treated with 6.5 mM metronidazole overnight were used as controls. Upon treatment, the larvae were transferred into fresh E3 medium.

### Microinjections of *S. aureus* into zebrafish larvae

Zebrafish larvae at 30 or 54 hpf were microinjected into the circulation with bacteria as previously described (Prajsnar *et al*., 2008). Briefly, anaesthetized larvae were embedded in 3% w/v methylcellulose and injected individually using microcapillary pipettes filled with the bacterial suspension of known concentration. Following infection, larvae were observed frequently up to 122 hpf, dead larvae removed and numbers recorded at each time point.

### Determination of *in vivo* bacterial burden

In order to recover bacteria from infected larvae, groups of zebrafish larvae were transferred individually (with 100 μl of E3) to microfuge tubes and mechanically homogenized using a micropestle (Eppendorf). The homogenates were serially diluted and plated out on BHI agar to determine *S. aureus* numbers. This procedure was repeated usually at 12 h time intervals following infection.

### Cy5-TSA coupled with L-plastin immunostaining

Larvae were fixed in ice cold 4% w/v paraformaldehyde in PBS overnight at 4°C. Fixed larvae were washed twice in PBS. Myeloperoxidase activity was detected by incubation in 1:50 Cy5-TSA : amplification reagent (PerkinElmer) in the dark for 10 min at 28°C. Larvae were washed in PBS-T [PBS plus 0.8% (v/v) Triton X-100] for 4 × 20 min and incubated in blocking solution [PBS-T with 10% (v/v) sheep serum] for 1 h at room temperature. After blocking, larvae were incubated overnight at 4°C in blocking solution containing rabbit anti-L-plastin antibody (a kind gift from Professor Paul Martin, University of Bristol, UK) at 1:500 final concentration, followed by washing four times for 20 min with blocking solution. Larvae were then incubated with secondary antibody (mouse anti-rabbit conjugated with Alexa Fluor 488, Invitrogen) for 2 h at room temperature. After washing (4 × 20 min) with PBS-T, larvae were washed in PBS and mounted on a microscopic slide in 80% (v/v) glycerol for microscopic examination.

### Microscopic observations of larvae

Live anaesthetized larvae were immersed in 1% w/v low melting point agarose solution in E3 medium and mounted flat on a transparent slide. Images were acquired using the TE-2000U microscope (Nikon) with a Hamamatsu Orca-AG camera. Image acquisition and processing were performed with Volocity (Improvision). Objectives of following parameters have been used: 2× Nikon Plan UW objective NA 0.06; 4× Nikon Plan Fluor objective NA 0.13; 10× Nikon Plan Fluor objective NA 0.30 and 60× Nikon Plan Apo oil objective NA 1.4. For fixed and stained larvae, images were acquired using the UltraVIEW VoX spinning disk confocal microscope (Perkin Elmer) with Olympus 10× UPlanSApo objective NA 0.40. No non-linear normalizations were performed.

### Mouse intravenous injections

Female BALB/c mice were purchased from Charles River (Margate, UK) and maintained in the animal facility, University of Sheffield using standard husbandry procedures. The 7–8 weeks old mice were inoculated intravenously in the tail with 100 μl of *S. aureus* SH1000 suspension in endotoxin-free PBS (Sigma) corresponding to 1 × 10^7^ cfu per mouse. Viable bacteria in the inoculum were counted by serial decimal dilutions to confirm the accuracy of the bacterial dose. Mice were individually monitored for up to 10 days before being sacrificed. Bacterial persistence in host tissues was evaluated by aseptically removing the kidneys, homogenizing them and performing viable bacteria counts after serial dilution in PBS. The number of colony-forming units was determined after 24 h cultivation on BHI agar plates.

### Model construction

To investigate the mechanisms underlying the high strain ratio variance, we developed a simple model that captures the key features of the interaction between *S. aureus* bacteria and the phagocyte population of a zebrafish larva during infection. In particular we assume that, extracellular bacteria are internalized by phagocytes which then control the infection. We used this model to investigate the hypothesis that a small subset of the phagocyte population are subverted by internalized bacteria, and permit the release of viable bacteria back into the host, thus providing a population bottleneck, commonly associated with distorted population patterns in ecology.

The model was constructed using a system of ordinary differential equations of a form commonly used to model infection dynamics (Tillett, 1992). We consider the host to comprise of a population of phagocytes. In the absence of infection they are considered to be naïve, here denoted by *M*, and are assumed to be long-lived relative to the duration of *S. aureus* infection and therefore have a negligible removal rate. A population of *S. aureus*, *S_0_*, is then introduced into the host, the *S. aureus* population, *S*, has an extracellular net growth rate, *r*. Encounters between a bacterial cell and a phagocyte are assumed to occur under a mass action process and result in internalization with a rate *β*, this can be considered as the rate at which an encounter between a bacterium and phagocyte results in ingestion of the bacteria by the phagocyte. Once a phagocyte has internalized a bacterial cell, it is termed ‘Effective’, *E*. Effective phagocytes continue to ingest bacteria but now have a finite lifespan, 1/*a*. We also assume that the population of phagocytes is maintained around a stable population size, *K*, and that, while there is no significant growth of total phagocyte numbers, if depleted, they will be replenished in a density dependent manner.

To model the subset of subverted phagocytes we introduce a population of phagocytes that are termed Infected, *F*. These are phagocytes that, on initial exposure to a bacterium, are manipulated such that instead of sequestering their contents, they release viable bacteria back into the host. Infected phagocytes occur with a probability *f* on the initial ingestion of a bacterium by a naïve phagocyte.

The dynamics of these processes can be described by the following equations, where *S* is the extracellular bacterial population, and *M* represents the populations of naïve phagocytes. After ingesting bacteria, naïve macrophages become Effective phagocytes with a probability 1-*f*, they have a lifespan of 1/*a* during which they continue to phagocytose bacteria and after which they successfully neutralize their contents. Infected phagocytes, *F*, arise from naïve phagocytes with a probability *f*, they are biologically indistinguishable from Effective phagocytes except that they eventually release their contents as viable bacteria back into the host, with an average burst size of *z*.


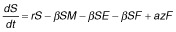



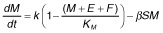



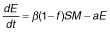



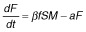


By definition, the deterministic model as written is unable to capture the variable nature of the data so the model was put into an individual based framework using a Markov chain formulation. The experimental results described in the paper demonstrate that the strains of *S. aureus* used (Tet^R^ and Ery^R^) are indistinguishable biologically, except in their growth on restrictive media. In a deterministic model, we would need to include additional variables to capture the dynamics of each strain; by using an individual based model it is easy to track the fate of each phagocyte, and its bacterial contents, individually and explicitly capture the dynamics of marked populations of *S. aureus*.

Using the parameters outlined in Table S1, we inoculate the host with an initial dose of two marked strains. We then follow the numbers of phagocytes of both subsets (Effective and Infected), the numbers of each strain ingested by each phagocyte and the numbers of both extracellular bacterial strain. An infection can be resolved when there are no viable bacteria left in the host, and a host is assumed to die due to infection with a probability proportional to its total bacterial load. The model can obtain mean infection sizes (in terms of total bacterial population) that are comparable with those seen in the experimental results; however, the experimental data show longer tails than is captured by the model, with more instances of very high total bacterial load and very low bacterial load. As validation we then used the model to look at the effect of changing the probability, *f*, of *infected* phagocytes occurring. Figure 4B shows the ratio, at time of death, of Tet^R^ to Ery^R^ obtained from the model. With an equal probability of effective and infected phagocytes arising (*f* = 0.5), very little variation is seen in the ratio of Tet^R^ to Ery^R^ and almost all hosts show evenly mixed final populations. Obvious variation in the Tet^R^ : Ery^R^ ratios at time of death are not seen until the probability of infected phagocytes arising are small (*f* < 0.16). When probability *f* = 0.03, equating to five infected phagocytes for every 145 effective ones, we still see high numbers of instances where the Tet^R^ : Ery^R^ strain ratio is evenly mixed but we also see a clear number of cases where extremely skewed ratios are observed (Fig. 4B) suggesting that the rate of infected phagocyte production is in the order of 3%. If the probability of infected phagocyte production drops too low (*f* < 0.01), the model predicts that the majority of cases result in extremely skewed Tet^R^ : Ery^R^ ratios, with very few instances where the strain ratio is evenly mixed. This suggests that a small but significant percentage of abnormal or permissive cells in the order of 3–10%, can be sufficient to give rise to the extreme variance in strain ratios observed in the experimental data.

### Statistical analysis

Survival experiments were evaluated using the Kaplan–Meier method. Comparisons between curves were made using the log rank test. For comparisons of myeloid cell or colony-forming units numbers between two groups (e.g. treated and non-treated) a two-tailed, unpaired Student's *t*-test was performed. A comparison between Tet^R^+/Ery^R^− and Tet^R^−/Ery^R^+ bacterial ratio distributions was done using the (non-parametric) Mann–Whitney test. To compare the variance of strain ratios recovered from embryos infected with a mixed co-inoculum of two *S. aureus* strains we used the two sample bootstrapped KS test to for the hypothesis that the probability densities for both samples were the same. Analysis was performed using Prism version 5.0 (GraphPad) or R, and statistical significance was assumed at *P*-value below 0.05.

## Supporting information

**Fig. S1.** A. Survival curve for zebrafish larvae (*n* = 20) injected at 30 hpf with 1200 cfu of the *S. aureus* strain indicated. *P* = 0.81 for JLA371 versus JLA513 strain.

B. Survival curve for zebrafish larvae (*n* = at least 20) injected at 30 hpf with 1200 cfu of the *S. aureus* strains indicated. *P* = 0.82 for JLA371/JLA513 mixture versus SH1000 strain.

C. Bacterial numbers (Tet^R^ and Ery^R^) recovered from LWT zebrafish larvae infected with 1200 cfu of mixed co-inoculum of *S. aureus*. Wild-type larvae were homogenized at the terminal stage of infection and bacterial numbers of both strains were determined on selective media.

D. Distribution of bacterial strain ratios (Tet^R^ to Ery^R^) recovered from terminally infected wild-type zebrafish larvae inoculated with a mixture consisting of 600 cfu of ‘Tet^R^+’ and 600 cfu of ‘Ery^R^−’. Larvae were homogenized at the terminal stage of infection and bacterial numbers of both strains were determined on selective media.

E. Distribution of bacterial strain ratios (Tet^R^ to Ery^R^) recovered from terminally infected wild-type zebrafish larvae inoculated with a mixture consisting of 600 cfu of ‘Ery^R^+’ and 600 cfu of ‘Tet^R^−’. Larvae were homogenized at the terminal stage of infection and bacterial numbers of both strains were determined on selective media.

**Fig. S2.** Microscopic observations of LWT (A), *irf8* (B) and *pu.1* (C) knock-down larvae injected with 1200 cfu of GFP-expressing *S. aureus* SH1000 at 30 hpf. Images were taken at 24 hpi using a 4× Nikon Plan Fluor objective NA 0.13. Ten Z slices were compressed into one ‘extended focus’ image. Typical embryos are shown for each condition.

**Table S1.** List of variables and parameters used in the mathematical model.

**Table S2.** List of kidneys and corresponding abscesses harvested from BALB/c mice 10 days after infection with a mixed Tet^R^ and Ery^R^ inoculum of *S. aureus*. Bacterial numbers of each strain recovered and their corresponding ratios are presented. Use of the number 1 indicates that the organ/abscess was sterile for this strain (1 was used instead of 0 for strain ratio calculation).

**Video S1.** *In vivo* time-lapse observation of *pu.1* knock-down larva (tail region) injected with a mixture consisting of 600 cfu of CFP- and 600 cfu of YFP-labelled *S. aureus* SH1000. Movie starts at 2 hpi and finishes at 18 hpi captured using 10× Nikon Plan Fluor objective NA 0.30.
