# Supplementary figures and images for: A privileged intraphagocyte niche is responsible for disseminated infection of Staphylococcus aureus in a zebrafish model
Source: Cell Microbiol. 2012 Jul 4;14(10):1600–19. doi: 10.1111/j.1462-5822.2012.01826.x (PMC3470706; doi:10.1111/j.1462-5822.2012.01826.x)

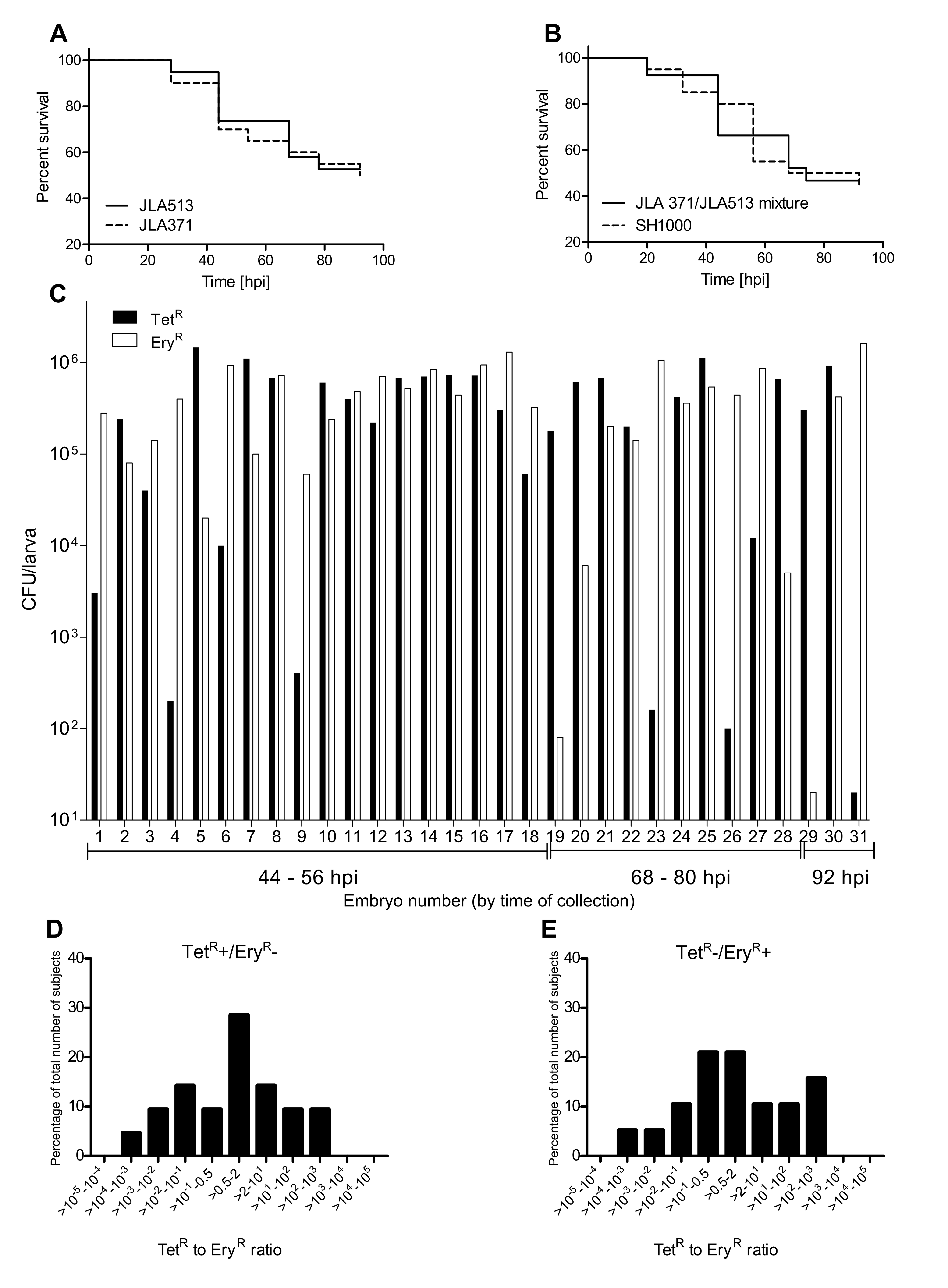

Supplement: Supplementary file 1 [file cmi0014-1600-SD1.tif]

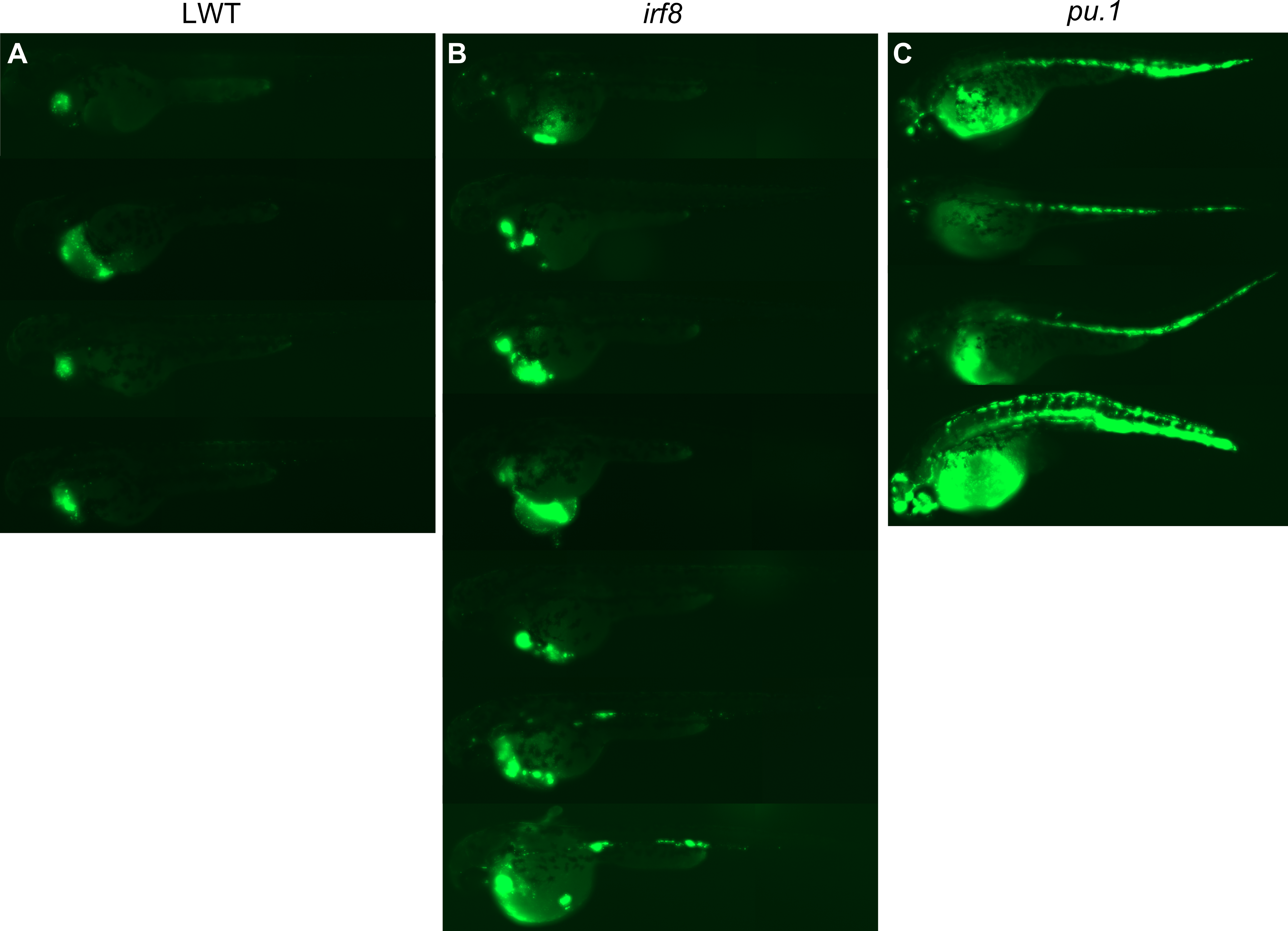

Supplement: Supplementary file 2 [file cmi0014-1600-SD2.tif]
