# Supplementary material for: A privileged intraphagocyte niche is responsible for disseminated infection of Staphylococcus aureus in a zebrafish model
Source: Cell Microbiol. 2012 Jul 4;14(10):1600–19. doi: 10.1111/j.1462-5822.2012.01826.x (PMC3470706; doi:10.1111/j.1462-5822.2012.01826.x)
Supplement: Supplementary file 3 [file cmi0014-1600-SD3.doc]

**Table S1.**

List of variables and parameters used in the mathematical model.

| **Symbol** | **Variables** | **Initial value** |
| --- | --- | --- |
| S | *S. aureus* population | 1200 |
| M | Naïve phagocytes | 150 |
| E | Effective phagocytes | 0 |
| F | Infected phagocytes | 0 |
|  | **Parameters** |  |
| *r* | Net growth rate of extracellular *S. aureus* | 1.5 |
| *β* | Coefficient of internalisation | 0.015 |
| *a* | Rate of decay of effective and infected phagocytes | 0.05 |
| *z* | Average contents of infected phagocyte | *[not used in IBM model]* |
| *k* | Rate of production of naïve phagocytes | 1.0 |
| *K* | Equilibrium population size of phagocytes | 150 |
| *f* | Probability of subversion of phagocyte to infected | 0.006-0.5 |
