# Supplementary material for: A privileged intraphagocyte niche is responsible for disseminated infection of Staphylococcus aureus in a zebrafish model
Source: Cell Microbiol. 2012 Jul 4;14(10):1600–19. doi: 10.1111/j.1462-5822.2012.01826.x (PMC3470706; doi:10.1111/j.1462-5822.2012.01826.x)
Supplement: Supplementary file 4 [file cmi0014-1600-SD4.doc]

## Table S2.

List of kidneys and corresponding abscesses harvested from BALB/c mice 10 days after infection with a mixed Tet^R^ and Ery^R^ inoculum of S. aureus. Bacterial numbers of each strain recovered and their corresponding ratios are presented. Use of the number 1 indicates that the organ/abscess was sterile for this strain (1 was used instead of 0 for strain ratio calculation).

| Kidney I | CFU (Tet^R^) | CFU(Ery^R^) | Tet^R^/Ery^R^ ratio |
| --- | --- | --- | --- |
| Abscess 1 | 1 | 6.7x10^5^ | 1.5x10^-6^ |
| Abscess 2 | 4.5x10^5^ | 2.6x10^5^ | 1.7 |
| Total (Abscesses) | 4.5x10^5^ | 9.3x10^5^ | 0.48 |
| Total (Whole kidney) | 4.5x10^5^ | 9.3 x10^5^ | 0.48 |
|  |  |  |  |
| Kidney II | CFU (Tet^R^) | CFU(Ery^R^) | Tet^R^/Ery^R^ ratio |
| Abscess 1 | 2.5x10^1^ | 1.6x10^5^ | 1.6x10^-4^ |
| Total (Whole kidney) | 1.3 x10^6^ | 2.2x10^5^ | 5.9 |
|  |  |  |  |
| Kidney III | CFU (Tet^R^) | CFU(Ery^R^) | Tet^R^/Ery^R^ ratio |
| Abscess 1 | 1 | 2.9x10^5^ | 3.4x10^-6^ |
| Total (Whole kidney) | 2.7x10^4^ | 2.0 x10^6^ | 1.3 x10^-2^ |
|  |  |  |  |
| Kidney IV | CFU (Tet^R^) | CFU(Ery^R^) | Tet^R^/Ery^R^ ratio |
| Abscess 1 | 5.8x10^5^ | 1 | 5.8x10^5^ |
| Total (Whole kidney) | 5.8x10^5^ | 1 | 5.8x10^5^ |
|  |  |  |  |
| Kidney V | CFU (Tet^R^) | CFU(Ery^R^) | Tet^R^/Ery^R^ ratio |
| Abscess 1 | 1.7x10^5^ | 1 | 1.7x10^5^ |
| Total (Whole kidney) | 1.9x10^5^ | 1 | 1.7x10^5^ |
|  |  |  |  |
| Kidney VI | CFU (Tet^R^) | CFU(Ery^R^) | Tet^R^/Ery^R^ ratio |
| Abscess 1 | 1.2x10^6^ | 7.5x10^2^ | 1.6x10^3^ |
| Total (Whole kidney) | 1.2x10^6^ | 7.5x10^2^ | 1.6x10^3^ |
|  |  |  |  |
| Kidney VII | CFU (Tet^R^) | CFU(Ery^R^) | Tet^R^/Ery^R^ ratio |
| Abscess 1 | 1 | 2.7x10^4^ | 3.7x10^-5^ |
| Total (Whole kidney) | 1 | 2.7x10^4^ | 3.7x10^-5^ |

| Kidney VIII | CFU (Tet^R^) | CFU(Ery^R^) | Tet^R^/Ery^R^ ratio |
| --- | --- | --- | --- |
| Abscess 1 | 1.2x10^6^ | 1 | 1.2x10^6^ |
| Abscess 2 | 1 | 7.9x10^5^ | 1.3 x10^-6^ |
| Total (Abscesses) | 1.2x10^6^ | 7.9x10^5^ | 1.5 |
| Total (Whole kidney) | 1.2x10^6^ | 7.9x10^5^ | 1.5 |
|  |  |  |  |
| Kidney IX | CFU (Tet^R^) | CFU(Ery^R^) | Tet^R^/Ery^R^ ratio |
| Abscess 1 | 2.6x10^5^ | 1 | 2.6x10^5^ |
| Total (Whole kidney) | 2.6x10^5^ | 1.2x10^6^ | 0.22 |
|  |  |  |  |
| Kidney X | CFU (Tet^R^) | CFU(Ery^R^) | Tet^R^/Ery^R^ ratio |
| Abscess 1 | 1.0 x10^5^ | 3.1x10^2^ | 3.3x10^2^ |
| Abscess 2 | 1 | 1.0x10^6^ | 1.0x10^-6^ |
| Total (Abscesses) | 1.0 x10^5^ | 1.0x10^6^ | 0.10 |
| Total (Whole kidney) | 1.0 x10^5^ | 1.0x10^6^ | 0.10 |
|  |  |  |  |
| Kidney XI | CFU (Tet^R^) | CFU(Ery^R^) | Tet^R^/Ery^R^ ratio |
| Abscess 1 | 2.3x10^5^ | 6.0x10^4^ | 3.8 |
| Total (Whole kidney) | 1.2x10^6^ | 1.7x10^5^ | 7.1 |
|  |  |  |  |
| Kidney XII | CFU (Tet^R^) | CFU(Ery^R^) | Tet^R^/Ery^R^ ratio |
| Abscess 1 | 5.5x10^4^ | 1 | 5.5x10^4^ |
| Total (Whole kidney) | 3.7x10^5^ | 3.0x10^4^ | 12 |
